# COVID‐19 vaccination and mental health in hospital workers

**DOI:** 10.1002/brb3.2382

**Published:** 2021-10-17

**Authors:** Genichi Sugihara, Nobuhide Hirai, Nori Takei, Hidehiko Takahashi

**Affiliations:** ^1^ Department of Psychiatry and Behavioral Sciences Tokyo Medical and Dental University Tokyo Japan; ^2^ Health Administration Center Tokyo Medical and Dental University Tokyo Japan; ^3^ Department of Psychosis Studies Institute of Psychiatry, Psychology, & Neuroscience, King's College London London UK

COVID‐19 vaccination has been shown to be highly effective in reducing new cases, hospitalizations, and deaths (Roest et al., 2021). Despite its effectiveness, low confidence in COVID‐19 vaccines in the public would cause vaccination hesitancy (Razai et al., 2021), which in turn may hinder the end of the pandemic. According to a survey (Imperial College London, 2021), Japan has the lowest confidence in COVID‐19 vaccines among 15 developed countries; only 47% of the respondents in Japan, while for instance 87% in UK, reported trust in COVID‐19 vaccines. However, this does not conform to healthcare professionals’ behavior. Japan commenced its vaccination with BNT162b2 (Pfizer‐BioNTech) in February 2021, and frontline health workers were first targeted for the vaccination as a governmental decision. As of June 16, 2021, the number of healthcare professionals who have received at least one dose of the vaccine was approximately 9.5 million (Prime Minister of Japan and His Cabinet, 2021), which far exceeds the number estimated by the Japanese Government, 4.7 million. This implicates that medical professionals in Japan have a high reliance on its vaccination.

The high rate of the vaccination among the healthcare professionals may merely reflect their sense of mission and duty in providing optimal medical care under the pandemic crisis. Healthcare workers have a fear of not only getting infected but also transmitting COVID‐19 to their families, colleagues, and uninfected patients (De Kock et al., 2021). Fighting against COVID‐19 with no security is a real threat, seriously harming work performance and mental health (Lu et al., 2021). In this context, the trust in COVID‐19 vaccines among medical professionals may convey additional benefits. One shot of vaccine in the frontline workers may contribute to the reduction of their stress‐related psychological breakdowns such as anxiety and depression.

This view was supported by our web‐based surveys on the mental state in hospital workers at our university hospital, in which 265 hospital workers (i.e., nurses, doctors, other medical professionals, researchers, and clerical staff; mean [SD] age, 42.8 [10.2]; 171 [64.5%] women) provided data before (January 13−28, 2021, around the peak of the third outbreak in Japan) and after the vaccination (April 19−30, 2021, the fourth outbreak). Anxiety and depression were assessed using the 7‐item Generalized Anxiety Disorder scale (GAD‐7) and the 9‐item Patient Health Questionnaire (PHQ‐9), respectively. The sample comprised 204 vaccinated (with at least one injection) and 61 nonvaccinated. We examined the influence of vaccination on mental state with a parallel process growth model. Because of the skewed outcome measures, maximum likelihood estimation with robust standard errors was employed.

We found a significant difference in the rate of change in both anxiety and depression (i.e., slope) between vaccinated and nonvaccinated groups, standardized parameter estimate of slope (*S*) = −0.504 (SE 0.125, *p* < .001) for GAD‐7, and *S* = −0.377 (SE 0.134, *p* = 0.005) for PHQ‐9 (Figure 1), implying that the hospital workers benefit from vaccination to reduce the risk of mental health problems. This was not explained by demographic variables such as age, gender, and occupation (i.e., medical vs. nonmedical staff). Of note, such effect was observed in the "outbreak" period, which linked to the risk for mental health problems. In addition, the vaccinated group had the higher level of both anxiety and depression on January 2021 compared with the nonvaccinated group, standardized parameter estimate of intercept (I) = 0.144 (SE 0.069, *p* = 0.037) for GAD‐7, and *I* = 0.173 (SE 0.068, *p* = 0.012) for PHQ‐9, suggesting that the group choosing to be vaccinated may have had more psychological distress, which is in line with the findings of a previous report (Kukreti et al., 2021). Again, the differences were not explained by the demographic variables.

**FIGURE 1:**
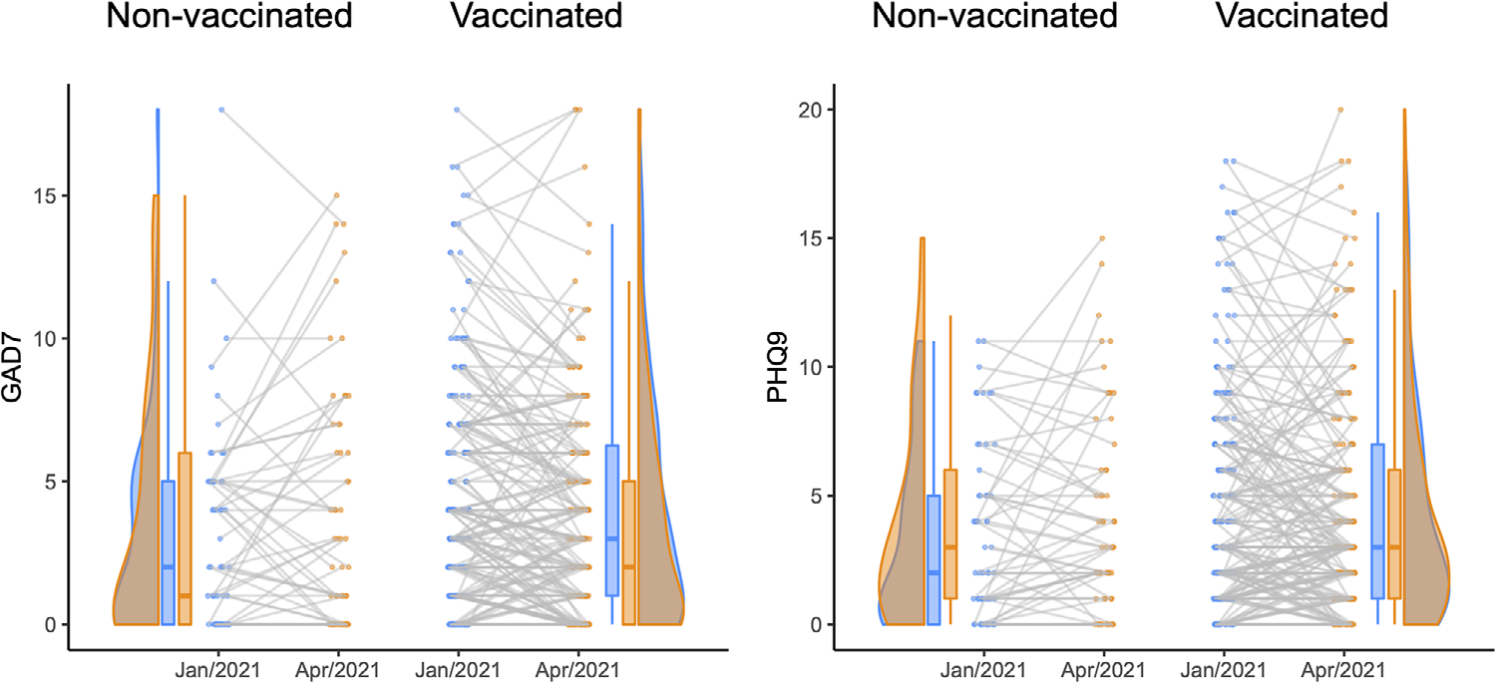
Changes of the levels of anxiety (GAD9) and depression (PHQ9) during January–April 2021 in vaccinated and nonvaccinated hospital workers

Our findings imply that COVID‐19 vaccination could have a protective effect in terms of well health in hospital workers, regardless of occupation, although the small sample size was a limitation of this study. COVID‐19 pandemic has caused overwhelming stress to not only medical professionals but also the general population. COVID‐19 vaccination would be promoted and accelerated in that the vaccination could be effective in not only protecting the infection but also in alleviating psychological distress.

## CONFLICT OF INTEREST

The authors declare no conflict of interest.

### PEER REVIEW

The peer review history for this article is available at https://publons.com/publon/10.1002/brb3.2382
